# (Noble Gas)_*n*_‐NC^+^ Molecular Ions in Noble Gas Matrices: Matrix Infrared Spectra and Electronic Structure Calculations

**DOI:** 10.1002/chem.202103142

**Published:** 2021-12-13

**Authors:** Yetsedaw A. Tsegaw, Hongmin Li, Lester Andrews, Han‐Gook Cho, Patrick Voßnacker, Helmut Beckers, Sebastian Riedel

**Affiliations:** ^1^ Anorganische Chemie Institut fur Chemie und Biochemie Freie Universitat Berlin Fabeckstrasse 34–36 14195 Berlin Germany; ^2^ Department of Chemistry University of Virginia Charlottesville Virginia 22904 USA; ^3^ Department of Chemistry Incheon National University 119 Academy-ro, Yeonsu-gu Incheon 22012 South Korea

**Keywords:** noble gases, cyanide cations, electronic structure calculations, IR probes, laser ablation, mercury

## Abstract

An investigation of pulsed‐laser‐ablated Zn, Cd and Hg metal atom reactions with HCN under excess argon during co‐deposition with laser‐ablated Hg atoms from a dental amalgam target also provided Hg emissions capable of photoionization of the CN photo‐dissociation product. A new band at 1933.4 cm^−1^ in the region of the CN and CN^+^ gas‐phase fundamental absorptions that appeared upon annealing the matrix to 20 K after sample deposition, and disappeared upon UV photolysis is assigned to (Ar)_*n*_CN^+^, our key finding. It is not possible to determine the *n* coefficient exactly, but structure calculations suggest that one, two, three or four argon atoms can solvate the CN^+^ cation in an argon matrix with C−N absorptions calculated (B3LYP) to be between 2317.2 and 2319.8 cm^−1^. Similar bands were observed in solid krypton at 1920.5, in solid xenon at 1935.4 and in solid neon at 1947.8 cm^−1^. H^13^CN reagent gave an 1892.3 absorption with shift instead, and a 12/13 isotopic frequency ratio–nearly the same as found for ^13^CN^+^ itself in the gas phase and in the argon matrix. The CN^+^ molecular ion serves as a useful infrared probe to examine Ng clusters. The following ion reactions are believed to occur here: the first step upon sample deposition is assisted by a focused pulsed YAG laser, and the second step occurs on sample annealing: (Ar)_2_^+^+CN→Ar+CN^+^→(Ar)_*n*_CN^+^.

## Graphical abstract

**CCSD(T) calculated Ng−C−N^+^ structures and observed frequencies**. The bond lengths on top are in Å unit, the natural charges in blue below atoms, and the observed C−N stretching frequencies (cm^−1^) in a matrix of noble gas on right side. Reactions of laser ablated Zn, Cd, and Hg atoms with HCN have provided an opportunity to observe photoionization of CN, giving a new band at 1933.4 cm^−1^ in the region of CN and CN^+^ gas phase fundamental absorptions. The absorption that appeared in annealing to 20 K and disappeared on UV photolysis has been assigned to (Ar)_n_CN^+^.

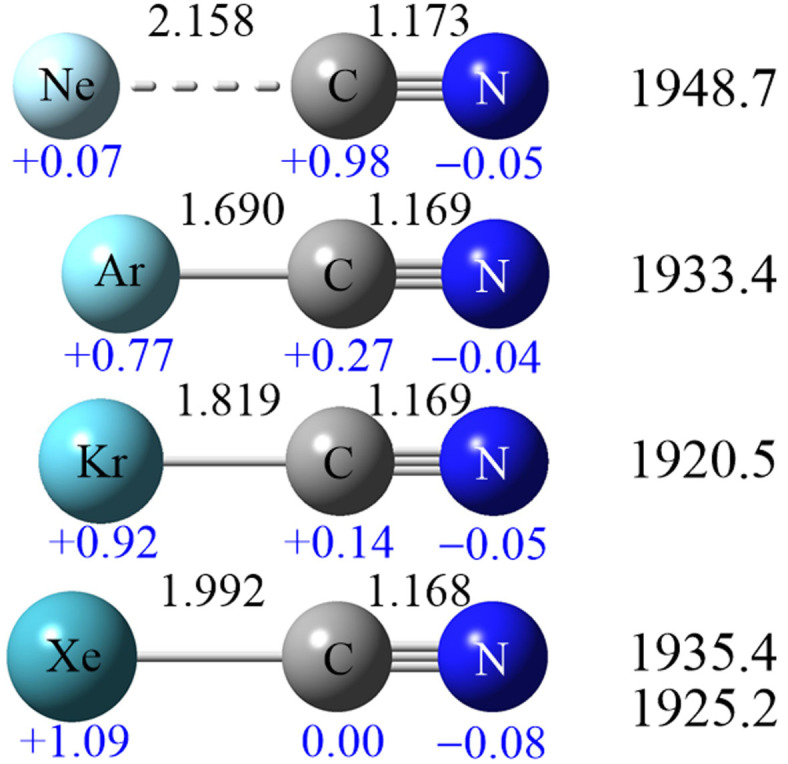

## Introduction

The matrix isolation technique was first developed to prepare, isolate, and examine new, reactive molecules by infrared spectroscopy. A good example is KrF_2_, which was first prepared by UV photolysis of a solid Kr and F_2_ mixture where the dissociation of F_2_ provided two F atoms to react directly with a nearby Kr atom.^[1]^ Much later the matrix‐isolation technique was adapted to obtain laser Raman spectra of trapped molecules where blue 488 nm continuous‐wave laser light was used to photolyse the F_2_ reagent and light scatter from the KrF_2_ molecule prepared in situ.^[2]^ In another study the 488 nm excited Raman spectrum of OF_2_ was examined in excess argon, and the Raman signals for OF_2_ decreased with time of exposure to the blue laser light while a new signal for the OF free radical photolysis product increased at 1029 cm^−1^.^[3]^ The matrix Raman measurement for OF radical was calibrated at 1028.9±0.5 cm^−1^ using argon emission lines, which is in excellent agreement with the matrix infrared measurement at 1028.6±0.3 cm^−1^ for the stretching fundamental of O−F prepared by the Li atom reaction with OF_2_ in excess condensing argon.^[3]^

A thorough investigation of the Group 12 metal atom reactions with HCN has been done under matrix isolation conditions,^[4]^ and the products were simple cyanides and isocyanides MCN and MNC, and their hydrides HMCN and HMNC, which were observed for the three metals. The mercury reactions were especially interesting because dental amalgam tooth filling material was employed as an ablation target for mercury.^[5]^ This is not possible with liquid mercury. Pictures of the mercury amalgam target are included at the end of this paper. Calculations using CCSD(T)/aug‐cc‐pVTZ (Zn) and aug‐cc‐pVTZ‐pp (Cd, Hg*)* showed the cyanides to be more stable (lower energy) than the isocyanides and this difference increased going down the periodic table in the group with about 20 kJ/mol for Zn, to 29 kJ/mol for Cd and 56 kJ/mol for Hg.^[4]^

**Figure 1:**
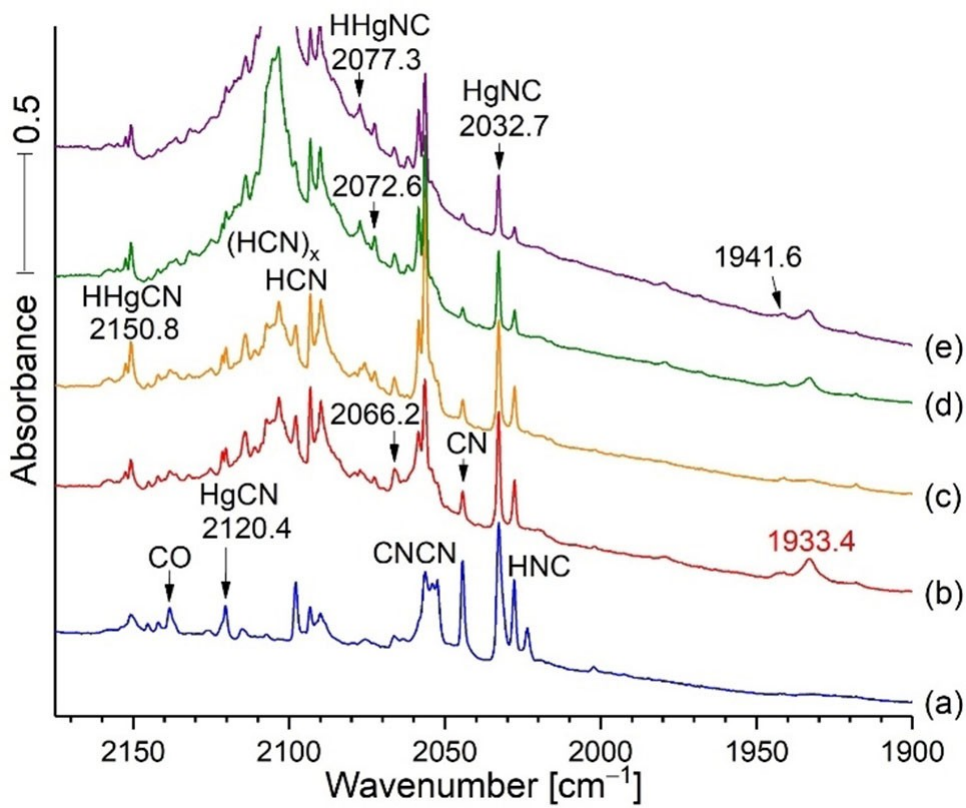
Infrared spectra of the reaction products of laser‐ablated Hg from a dental amalgam target with 0.2 % HCN under argon during condensation at 5 K. Note that the sample was exposed to Hg ablation plume radiation (intense bright white light) during the deposition (spectrum a). Spectrum a followed co‐deposition of the reagents for 2 h; spectrum b was recorded after annealing to 20 K and cooling back to 5 K, a new band was obtained at 1933.4; spectrum (c) was taken after 20 min of full medium‐pressure mercury arc photolysis; the 1933.4 absorption was destroyed after photolysis (spectrum c), but restored in part after annealing to 30 and 35 K (spectra d and e). The new mercury‐bearing reaction products HgCN and HgNC, and their hydrides are identified in spectrum e.^[4]^ Figures 2–4 compare the analogous spectra with H^13^CN and HC^15^N (99 % enriched); here annealing to 20 K shifted the new band to 1892.3 and 1908.6 cm^−1^, respectively.

The C−N stretching frequencies for the cyanides decreased from 2162.2 for ZnC−N to 2140.9 for CdC−N to 2120.4 cm^−1^ for HgC−N. (In this manuscript frequencies will always be given in cm^−1^ units). However, the M−N−C stretching frequencies decreased from 2074.6 for ZnNC to 2069.7 for CdNC and to 2032.7 cm^−1^ for HgNC. Notice that the isocyanide frequencies are lower than the cyanide values and they also decreased on going down the periodic table in the Group 12 family. The isocyanide frequencies are almost an order of magnitude more intense (calculated, CCSD) than the corresponding cyanide values, which arise from substantial bond polarity in the isocyanides that is absent in the cyanides.^[4]^ Figure 1 spectra for the Hg reaction with HCN shows that the HgNC band at 2032.7 cm^−1^ for HgNC is about five times stronger than the 2120.4 band for HgCN.^[4]^

When noble gas (Ng) atoms react in noble gas matrices, new compounds like KrF_2_ can be prepared.^[1]^ Similarly the UV photolysis of Cl_2_ in solid xenon has also produced XeCl_2_ ^[6]^ including blue laser photolysis during recording the laser Raman spectrum shifted to 254.6 cm^−1^.[^2^, ^3^]

To assist in the search for noble gas compounds in the interstellar medium vibrational frequencies and spectroscopic constants for three stable noble‐gas‐containing molecular ions NeCCH^+^, ArCCH^+^ and ArCN^+^ have been calculated.^[7]^ The latter is of particular interest to us here. The ionization of large homo and heterogeneous clusters generated in C_2_H_2_/Ar gas expansions including the argonium ion, ArH^+^ have been investigated.^[8]^ Argon is often considered as a passive probe that can be added or subtracted to study the structure of a solvated species without much perturbation.^[9]^ Reactions of noble gas dimer cations such as Ar_2_^+^ with hydrogen gas have been studied at 200 K, and H_2_^+^ is the major product.^[10]^ Finally the argonium molecular ion ^36^ArH^+^ has been identified in the Crab Nebula through rotational emission spectroscopy; this was the first noble gas molecular ion to be detected in interstellar space.^[11]^ It is interesting to find that the lighter ^36^Ar isotope is dominant in this emission spectrum, whereas in our terrestrial region argon‐40 (99.6 %) is the major isotope.^[12]^

The hydrogen cyanides were synthesized by reacting KCN with dilute H_2_SO_4_ and drying the HCN gas product by trap to trap distillation.^[13]^ The H^13^CN was made from K^13^CN (99 % ^13^C) in the same way, and likewise for HC^15^N (96 % ^15^N).

**Figure 2:**
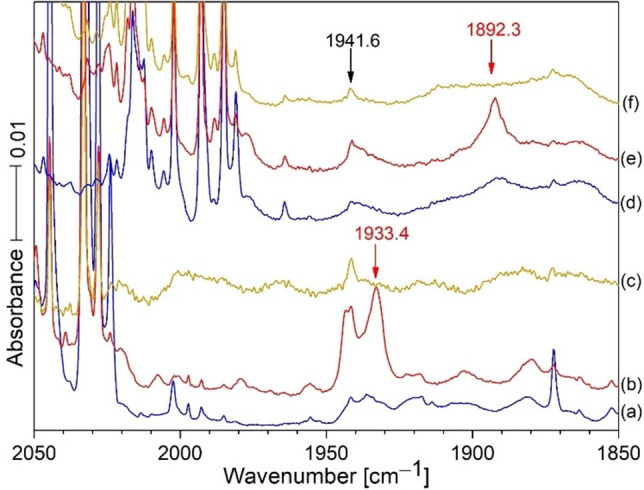
The bottom three scans are from HCN and the top three are from H^13^CN reactions both co‐deposited with Hg onto a 5 K substrate (CsI). The weak sharp band at 1941 cm^−1^ arises from our amalgam sample: It clearly does *not* track with the 1933.4 and 1892.3 bands on photolysis, scans (c) and (f). The last very strong band on the left is due to ^12^CN, and ^13^CN is at 2002.2 cm^−1^ in scan (a) using the HCN sample, which has 1 % ^13^CN in natural abundance. The very strong band straight above in scan (d) using 99 % ^13^C‐enriched HCN, is also due to ^13^CN.The red scans (b) and (e) show major growth for the sharp bands at 1933.4 and 1892.3 cm^−1^ upon annealing to 20 K. These bands give a 422.2 ^13^CN shift and a 1.02123 12/13 isotopic frequency ratio.^[4]^ The band at ca. 1873 is probably an impurity in the amalgam, as it shows no HCN isotopic shift.

This HCN spectrum and another one using H^13^CN, Figure 2, reveal the broad band at the same position on deposition as the sharp band and approximately the same band areas for the sharp and broad bands, which give way on annealing to 20 K to form the sharper bands at 1933.4 and 1892.3 cm^−1^. We presume that the broad versus sharp band distribution depends upon physical effects during deposition such as gas spray on rate and cold window temperature with the broad band favored by quicker non‐equilibrium freeze trapping conditions and the sharper band after annealing to obtain more stable equilibrium conditions. Band sharpening is a characteristic of early sample annealing cycles to attain more uniform and stable matrix site configurations. Figure 2 shows the same annealing behavior for the two precursor isotopes with broad band decrease and sharp band increase for the new products: the broad band is due to CN^+^ in quickly formed random Ar distribution and the sharp bands are due to complete more stable argon distributions around the CN^+^ cation centers.

**Figure 3:**
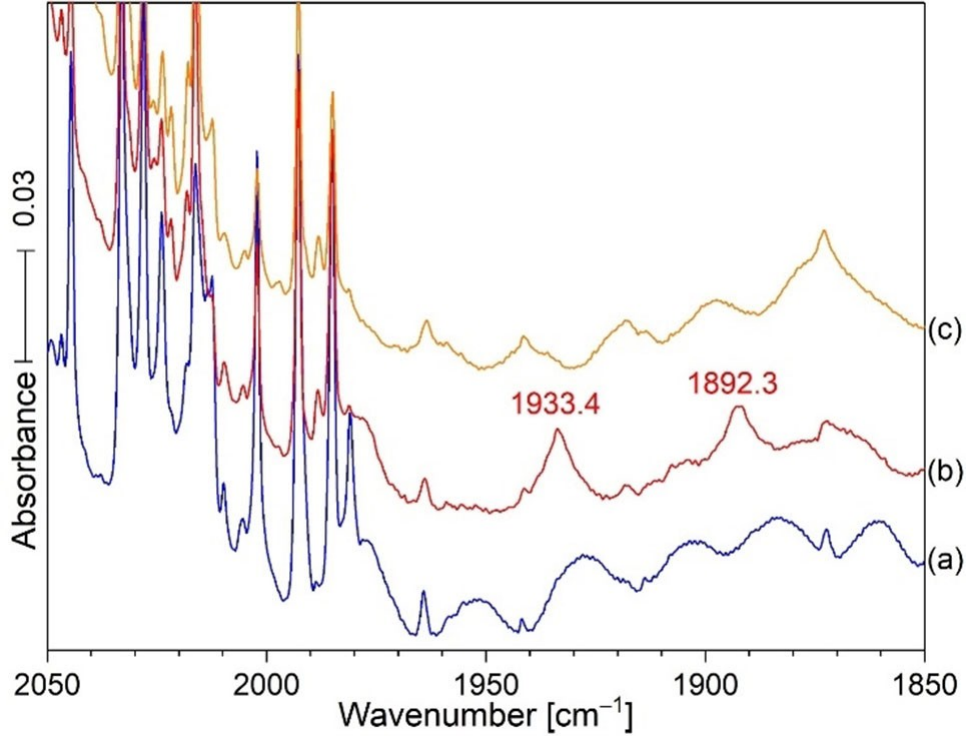
Infrared spectra from mixed H^12^CN/H^13^CN in the same sample (0.2 % each in an argon isotopic precursor experiment with ablated Hg). a) After sample deposition for 2 h; b) after annealing to 20 K, and c) after UV photolysis for 20 min. Notice the increase upon annealing of the two sharp red bands at 1933.4 and 1892.3 cm^−1^ at the expense of the broad bands underneath and their removal by UV light as before.

Notice that the new band at 1933.4 cm^−1^ did not appear on sample deposition other than as a broad weak band at 1933 cm^−1^ because of its photodissociation by the mercury target ablation emissions that produced the next reagents (probably CN and CN^+^). This new sharp product probably forms from the broad band on annealing to 20 K and cooling back to 5 K (Figure 1). Notice in Figure 1 (c), that external Hg arc photolysis almost destroyed the 1933.4 band, which explains why ablation plume radiation would do likewise, and annealing without light is necessary for the sharp product band to form. It is important to notice that the 2044.7 band for CN decreased on annealing to 20 K, while the band at 1933.4 cm^−1^ in the red spectrum, increased. Photolysis with Hg arc UV almost destroyed the sharp band at 1933.4 cm^−1^ in (c), but annealing to 30 K (d) reproduced about half of the original 1933.4 band, and a final annealing to 35 K slightly increased this feature. These new bands (Figure 2, H^13^CN) reveal a shift of 41.1 cm^−1^ and a 12/13 isotopic frequency ratio of 1.02171. An experiment with a 50 : 50 mixture of HCN/H^13^CN gave the spectra in Figure 3. Broad unknown bands were observed on sample deposition but annealing to 20 K produced a doublet of strong sharp red bands at 1933.4 and 1892.3 cm^−1^ read by the computer for the single isotopic precursors. A final UV photolysis destroyed these red bands as before.

**Figure 4:**
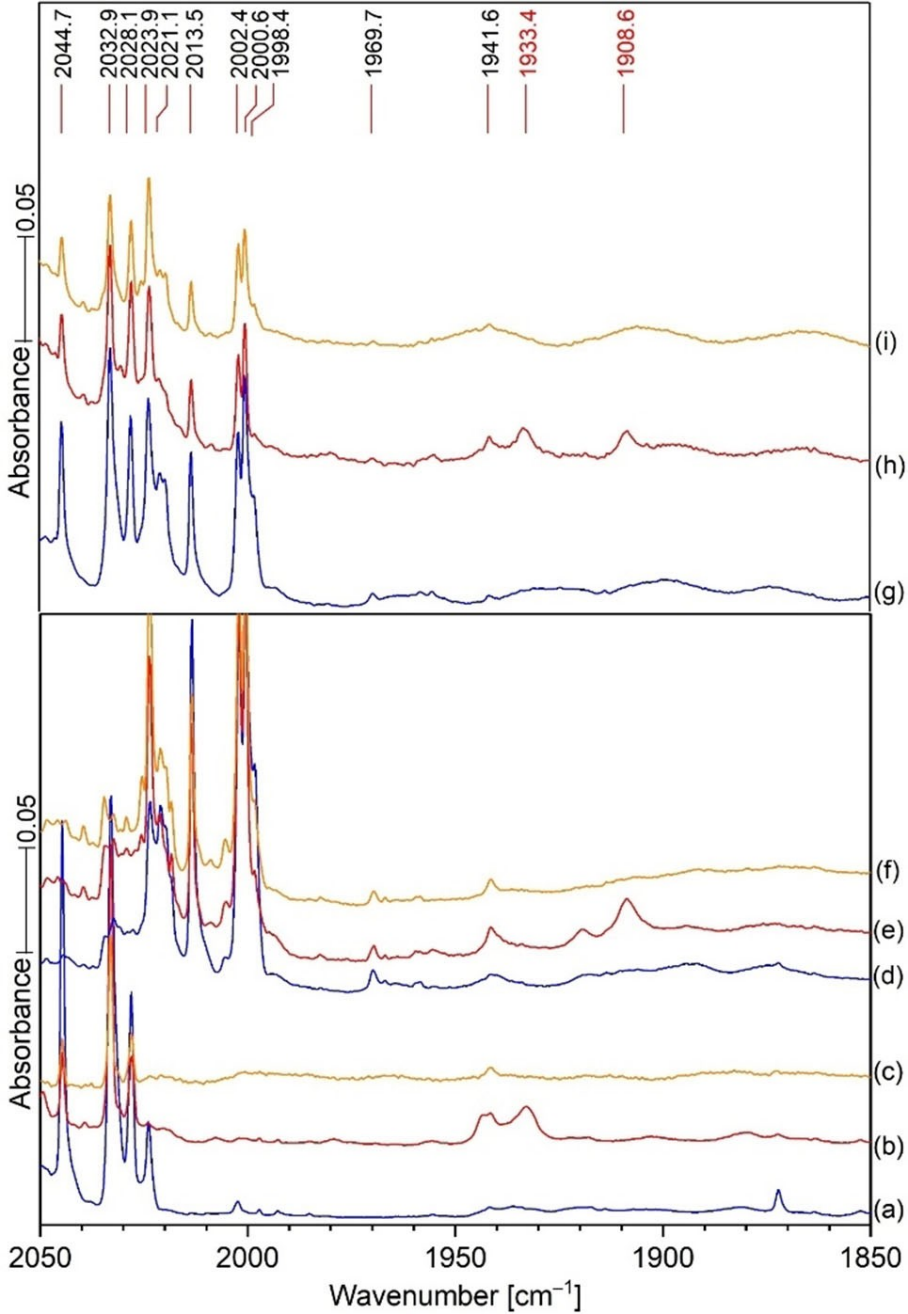
Comparison of the mixed HCN and HC^15^N isotope reaction spectra (top 3 scans). Spectra after sample deposition for 2 h (g), after annealing to 20 K (h), and after UV photolysis by Hg arc lamp (i). New bands at 1933.4 and 1908.6 cm^−1^ in spectrum (h). They are seen after the same treatment for each precursor isotope separately using the same color code: 1933.4 cm^−1^ for HCN reaction (b). The HC^15^N reaction with Hg under argon gave a new band at 1908.6 cm^−1^ after annealing (e). Clearly the mixed isotopic sample provided the same new bands as the separate isotopic precursor reactions.

IR spectra of the reaction products from laser ablated Hg co‐deposited with 0.2 % H^13^CN in argon at 6 K are in Figure 2, top set. Spectra after deposition for 120 min (a), annealing to 20 K and cooling back to 6 K (b), and after 20 min of irradiation with a medium‐pressure mercury arc (c). It is important to notice the broad bands underneath the two product bands at 1933.4 cm^−1^ in the bottom set of scans, and 1892.3 cm^−1^ in the top blue scans after sample deposition. The areas of the broad bands underneath are about the same as the sharp bands above. Annealing to 20 K allows these broad bands to sharpen without significant increase in the amount of these principal products. This is common behavior for the effect of annealing as the matrix often freezes before the most stable matrix packing arrangement is achieved, and annealing allows for atomic movement and more stable arrangements of matrix atoms and sharper bands to follow this process. The bottom set of scans compares additional spectra using the HCN precursor in Figure 2. This clear carbon isotopic doublet indicates that the 1933.4 absorption involves a single carbon atom. Similar experiments were done with HC^15^N and with 14/15 mixtures (Figure 4); these revealed a new 1908.6 band for HC^15^N and a doublet at 1933.4 and 1908.6 cm^−1^ for the HCN and HC^15^N bands together in the same sample like in Figure 3. This doublet shows that the new 1908.6 band involves a single N atom in a new matrix isolated chemical species. Similar Zn and Cd spectra were carefully examined, and there is *no* counterpart for this 1933.4 band in the spectra produced by the lighter Zn and Cd metals. This might provide a clue for product identification. The resonance emissions of Hg are higher energy than those for Cd and Zn.^[14]^ Finally the labeled absorptions in Figure 1, left side, have been identified in our previous work.^[4]^

As in Figure 3 for the carbon isotopes, the spectra in Figure 4 for nitrogen isotopes demonstrate that a single N atom is observed in this new product which must then be a CN species. It was observed at 1908.6 cm^−1^. The nitrogen 14 to 15 shift is 24.8 cm^−1^ and their ratio is 1.01299. The high‐resolution gas‐phase spectra for these two isotopes (2000.759 and 1970.321 cm^−1^)^[15]^ reveal a slightly larger 30.4 cm^−1^ shift and 1.015448 14/15 ratio than observed for the argon matrix product bands. These isotopic counterparts have identical behavior on annealing and photolysis of the sample.

What do the position and isotopic data for the 1933.4 cm^−1^ band tell us about it? The observation of only two new product bands for the mixed isotopic reactions, which are almost the same as the single isotopic products, show that the 1933.4 absorbing species contains single C and N atoms and thus it is surely a CN species. The neutral radical can be dismissed as it is observed at 2044.4 in solid argon and at 2046.16 cm^−1^ in the gas phase.^[16]^ It is unreasonable to expect an essentially nonpolar molecule to shift 113 cm^−1^ in an argon matrix. The Jacox group assigned the CN^−^ anion at 2053.1 cm^−1^ in solid neon and this neon matrix shift to argon would be at least 120 cm^−1^ which is not likely.^[17]^ Finally the gas‐phase band origin for CN^+^ is 2000.8 cm^−1[15]^ and this could matrix shift 67 cm^−1^ to the 1933.4 cm^−1^ value in solid argon.

**Figure 5:**
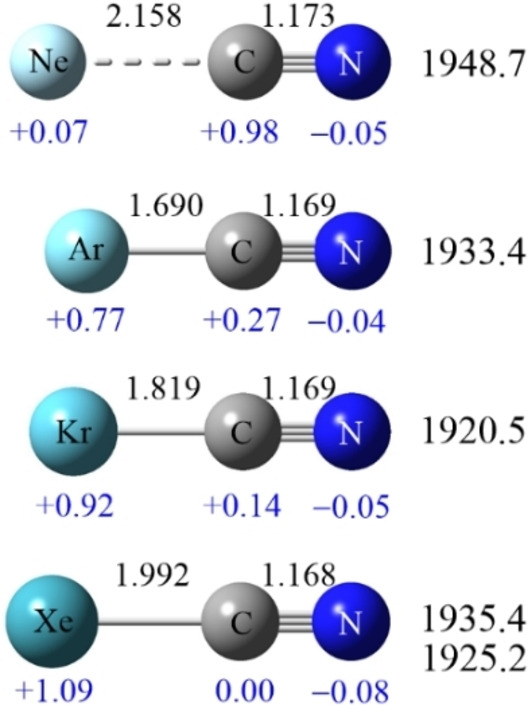
CCSD(T) calculated Ng−C−N^+^ Structures: bond lengths [Å] are shown on top, natural charges below atoms, and observed C−N stretching frequencies [cm^−1^] in a matrix of Ng on the right.

The high‐resolution measurement for the C−N fundamental vibration of CN is 2046.1 cm^−1 [18]^ and the corresponding values for ^12^CN^+^ and C^15^N^+^ are 2000.759 and 1970.321 cm^−1^ respectively (gas discharge).^[15]^ Application of the isotope invariant model gave 1959.4 cm^−1^ for the unobserved ^13^CN^+^ isotopic species in the gas phase. From the position (wavenumber value) this new CN matrix isolated species is most likely CN^+^. This would require a matrix shift from 2001 to 1933.4 cm^−1^ for the CN^+^ cation in solid argon. We examined the isotopic shift and ratio for the 1933.4 band and found the ^13^C shift and 12/13 isotopic frequency ratio are 41.1 cm^−1^ and 1.02171, which are very close to the 42.2 cm^−1^ and 1.02108 values observed for the CN radical trapped in these experiments (Table 1). Next the ^15^N data for the 1933.4 band is slightly lower than the gas‐phase band values, and the shift is 24.4 cm^−1^ and the 14/15 ratio is 1.01278. This data tells us that we have a diatomic mechanical CN subunit for the 1933.4 absorber and its ^13^C shift almost matches the value for the C−N diatomic molecule in the gas phase, but the ^15^N data are not as close suggesting that the N vibration is slightly perturbed and does not move as much as it would if it were in a diatomic relationship with C. The isolated Ar‐CN radical shows that the C atom is largely protected in this product (Figure 5) but the N on the end is exposed and subject to additional weak interactions with the matrix atoms.

**Table 1:** Calculated and observed frequencies (cm^−1^ in Ar or given matrix).

Species	Obs	B3LYP		CCSD(T)				
		Freq. (C−N)	Δ*E* [kJ/mol]	Freq. (C−N)	*r*(Ng−C)	*r*(C−N)	Δ*E* [kJ/mol]	Natural charges
CN	2044.4	2150.6 (20)		2109.4		1.174		+0.45, −0.45
in Ar								
CN^+^	1933.4	2077.3 (2)		1961.6		1.167		+1.04, −0.04
in Ar								
Ne⋅⋅⋅CN^+^	1947.8	2237.3 (4)	−221.2	1979	2.1578	1.173	−12.1	+0.07, +0.98, −0.05
in Ne								
Ar⋅⋅⋅CN^+^	1933.4	2317.8 (3)	−349.5	2200.7	1.69	1.169	−219.6	+0.77, +0.27, −0.03
in Ar								
Kr⋅⋅⋅CN^+^	1920.5	2309.4 (8)	−436.3	2201.5	1.8188	1.169	−312.8	+0.91, +0.13, −0.05
in Kr								
Xe⋅⋅⋅CN^+^	1925.2	2298.5 (15)	−544.1	2196.7	1.9913	1.168	−424	+1.09, −0.00, −0.08
in Xe^[a]^								

[a] for 1 % Xe in 99 % Ar: 1932.6.

Figure 5 compares structures for the four Ng cyanides.

The longest,1.173 Å, weakest Ng−C bond is with Ne, also with the highest N−C frequency,1948.7 cm^−1^. In this case the N−C linkage is a triple bond almost the same as the CN radical itself with a 2044.4 cm^−1^ frequency and 1.174 A bond length.

The Ar, Kr, and Xe complexes have nearly the same 1.68, 1.69 A C−N distances and 1920 to1935 frequencies.

**Figure 6:**
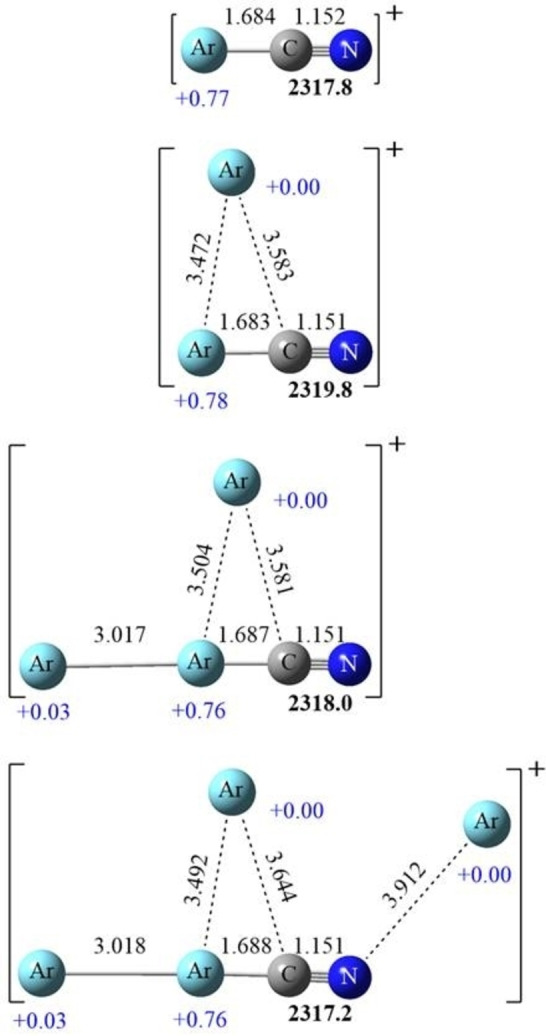
B3LYP calculated structures for Ar_*n*_−CN^+^ ion molecules: the bold number is the N−C frequency, bond lengths are given above the bonds and natural charges below the argon atoms.

What is the likely bonding situation for a CN^+^ cation in solid argon? In the gas phase, CN^+^ (with one less bonding electron) is 46 cm^−1^ lower than the CN radical. Therefore, in the argon matrix CN^+^ should be about 46 cm^−1^ below CN (2044.4 cm^−1^), which would provide a 1998 estimate for CN^+^ in solid argon depending on the effect of the matrix interaction. Table 1 gives this as 219.6 kJ/mol exothermic and Figure 6 shows that additional Ar atoms may alter this by up to 2 cm^−1^.

**Figure 7:**
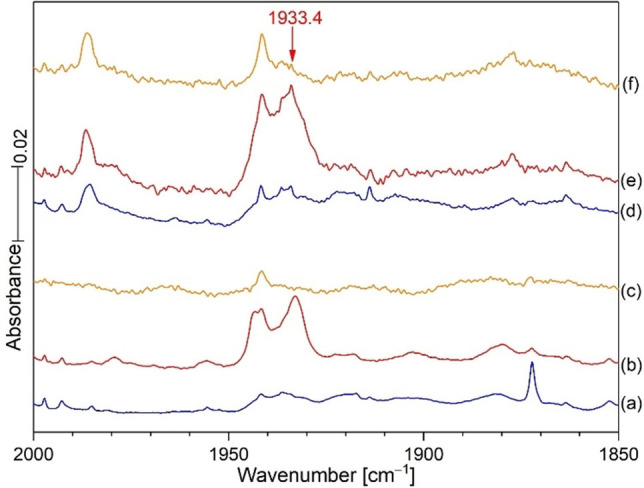
IR spectra of the reaction products from laser‐ablated Hg co‐deposited with 0.2 % HCN (spectra a–c) and 0.2 % HCN+0.05 % CCl_4_ (spectra d–f) in argon at 6 K. Spectra after deposition for about 2 h (blue), annealing to 20 K and cooling back to 6 K (brown), and 20 min of irradiation with a full‐medium pressure mercury arc lamp (yellow).

A trace of CCl_4_ was added as an electron trap in selected experimental samples as a diagnostic for charged species. In these cases, extra electrons can be captured by added CCl_4_ molecules and form the Cl^−^ anion and CCl_3_ radical along with some CCl_3_^+^. This condition allowed the survival of more isolated cations and reduced the number of isolated anions because fewer electrons were available to form new molecular anions or to neutralize isolated cations. Figure 7 shows that the 1933.4 band has about twice the area with CCl_4_ added to the sample mixture to capture photoelectrons produced in these samples. In addition, an ultra‐strong band for the CCl_3_ radical was observed at 898 cm^−1^ and the CCl_3_^+^ cation appeared at 1037 cm^−1^: these bands were photosensitive with visible and UV light from the mercury arc lamp.^[19]^ This method for charge identification was developed for the Rh carbonyl system with CO^+^ and both RhCO^+^ and RhCO^−^ in the sample owing to the laser ablation of Rh and Rh^+^ and photoionization of CO during sample deposition. The Rh ablation process was capable of the photoionization of CO in the argon sample.^[19]^

The argonium molecular ion ArH^+^ has been observed through two rotational emission lines from the Crab Nebula and it is the first noble‐gas‐containing species to be observed in the interstellar medium.^[11]^ Bondybey and Pimentel deposited hydrogen and excess argon through a microwave discharge onto a 14 K surface and observed a new IR absorption for this sample at 904 cm^−1^, and with deuterium this treatment gave instead a 644 absorption. The two bands have a 904/644=1.404 ratio which is appropriate for an Ar−H vibration. These workers next employed krypton and found an 852/607=1.404 ratio as well for the Kr analogues. Then ^36^Ar substitution produced a 0.2+/−0.1 cm^−1^ blue shift from natural ^40^Ar and confirmed the identification of argon in a new H−Ar vibration assigned then to an interstitial H atom isolated in octahedral sites in the solid argon lattice.^[8]^

Milligan and Jacox made similar argon matrix depositions and absorptions but preferred the positive ion identification as (Ar)_*n*_H^+^.^[9a]^ Andrews, et al. compared microwave discharge experiments using two different quartz tube geometries, the first with a coaxial 2 mm orifice in the tube end directed to the cold window so that the VUV(vacuum UV) light in the discharge irradiated the sample collection window during deposition, and the second with the *orifice in the left side of the discharge tube one cm from the closed front end* ^[10]^ where the hydrogen/argon mixture could reach the cold window but no VUV light from the *discharge body could reach* the sample collection window. These differences were interpreted to mean that coaxial discharge VUV radiation was needed to produce H^+^ and without that light only neutral Ar and H atoms flowed onto the cold window off axis and H could only do reactions such as combine with trace O_2_ to make the radical HO_2_ without the energy required to form positive ions.^[10]^ A picture of the rich Zr ablation plume is illustrated in ref. [20].

The pulsed laser‐ablation deposition experiment is similar to the coaxial discharge investigation as laser ablated excited bulk metal and its atoms and their resonance radiation can do photochemistry on the molecules included with the matrix gas such as HCN. With this understanding the Hg ablation produces higher energy radiation than its lighter Group12 members Cd and Zn, and Hg provides more photochemistry in the condensing matrix.[^14^, ^19^, ^21^] Also it is known that the reaction of noble gas dimer cations, that is, Ar_2_^+^,with H_2_ gas at 200 K produces an Ng atom and H_2_^+^.^[22]^ We suggest that this reaction with CN or HCN under the high energy “discharge” conditions of laser ablation will likewise produce CN^+^, which is the core for the Ng complexes formed here. In addition, doping the sample with the electron trapping molecule CCl_4_ serves as a diagnostic for cations as trapping electrons from the deposition process increase the fraction of cations that can survive as described previously^[19]^ and illustrated in Figure 7. The final equation given below summarizes the important new ion molecule reaction that is observed here.

$$\mathrm{Ar}{}_{2}{}^{+}+\mathrm{CN}\to \mathrm{Ar}+\mathrm{CN}{}^{+}\to \mathrm{Ar}{}_{n}-\mathrm{CN}{}^{+}$$

Therefore with light from noble‐gas‐discharge experiments similar to those done five decades ago we produce Hg ablation photochemistry of HCN including CN and probably CN^+^ that survive UV photolysis, and on annealing CN^+^, which like H^+^, CN^+^ supports 1, 2, 3 or 4 argon atoms in complexes analogous to their proton predecessors Ar_*n*_−H^+^.[^8^, ^9^, ^10^]

**Figure 8:**
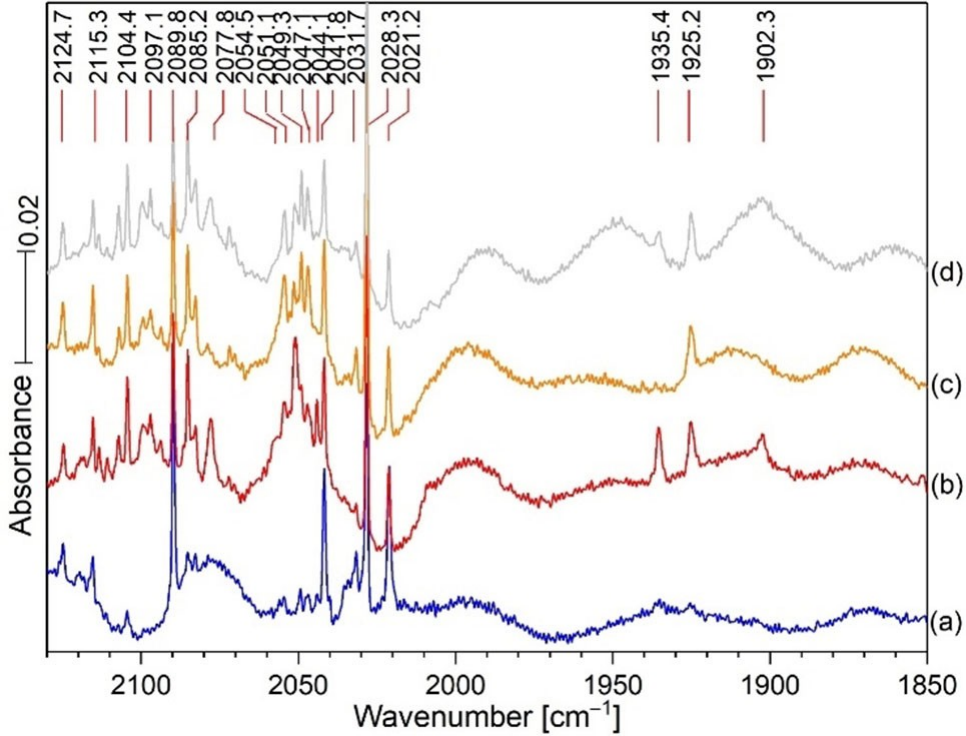
IR spectra of the reaction products from laser ablated Hg co‐deposited with 0.2 % HCN in a xenon matrix. Spectra after a) deposition for 60 min at 20 K and measurement at 6 K, b) annealing to 45 K and cooling back to 6 K, c) 20 min of irradiation with a full medium‐pressure mercury arc lamp, d) annealing to 50 K and cooling back to 6 K. The bands at 1935.38 and 1925.17 are probably matrix site splitting for the Xe−C−N^+^ product.

**Figure 9:**
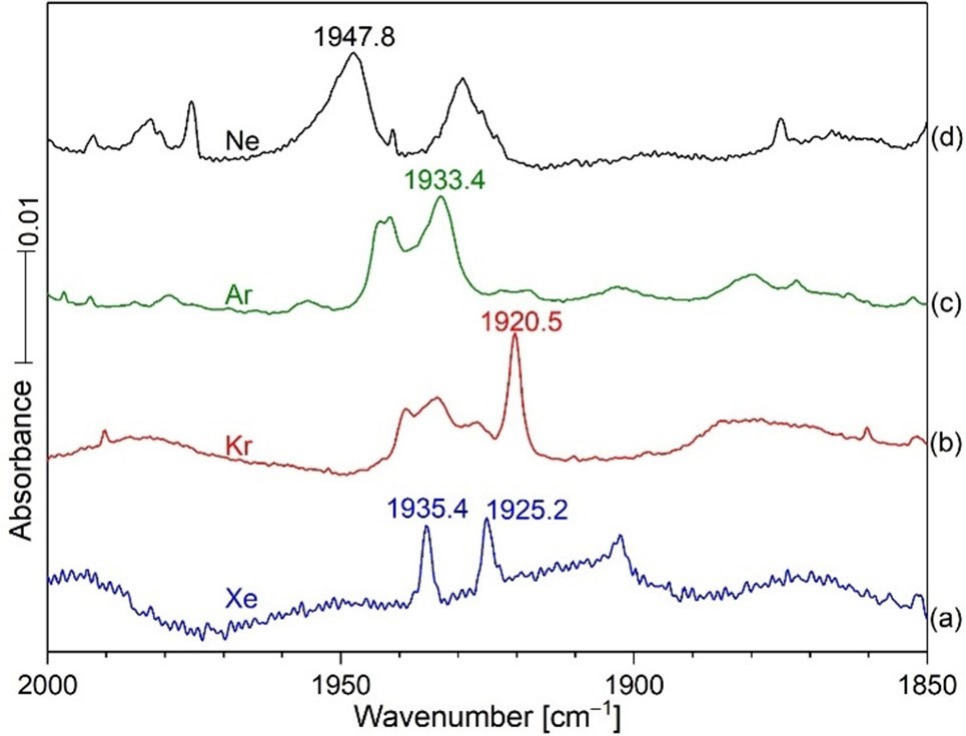
Infrared spectra of four noble gases each co‐deposited with HCN and Hg at 6 K.

**Figure 10:**
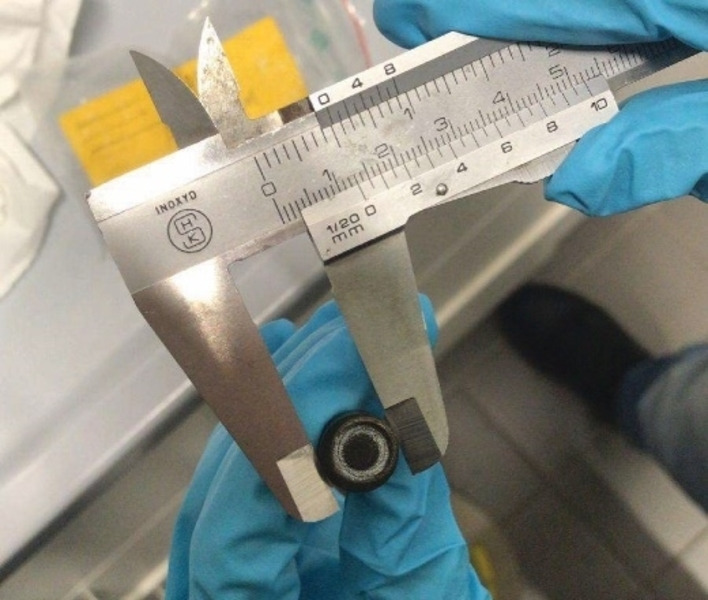
Mercury amalgam target.

Figures 8 and 9 show spectra of Hg and HCN reaction products in solid xenon and a comparison with these products in the four noble gas matrixes. The C−N^+^ frequency is higher for the lighter Ne Ng. A picture of the mercury amalgam target used in these experiments is presented in Figure 10. This target is rotated during the ablation process, and it continues to produce unique mercury bearing molecules for matrix infrared spectroscopic investigations.

## Conflict of interest

The authors declare no conflict of interest.
